# First record of *Arthromelodes* Jeannel in China, with description of a new species (Coleoptera, Staphylinidae, Pselaphinae)

**DOI:** 10.3897/zookeys.744.23318

**Published:** 2018-03-19

**Authors:** Zi-Wei Yin

**Affiliations:** 1 Department of Biology, College of Life and Environmental Sciences, Shanghai Normal University, Shanghai, China

**Keywords:** *Arthromelodes*, China, new species, Pselaphinae, Taiwan, taxonomy

## Abstract

A new species of *Arthromelodes* Jeannel, *A. choui* **sp. n.**, is described from northern Taitung, Taiwan, representing the first record of the genus in China. The species is distinctive, and may be readily separated from its congeners by the unique structures of male tergite 1(IV), and the aedeagus.

## Introduction

The genus *Arthromelodes* Jeannel of the pselaphine tribe Batrisini is a group of modest size containing 21 species and subspecies distributed in Japan (20 spp.) and Vietnam (1 sp.) (Jeannel 1954, 1957–1958; Nomura 1991; Arai 2002). This genus was included in Jeannel’s fifth division of the Batrisina (Jeannel 1958, 1959) and Nomura’s ‘*Batrisocenus* complex of genera’ (Nomura 1991) of Asian Batrisini, where members share a median and a pair of lateral longitudinal sulci, and a complete transverse antebasal sulcus on the pronotum, the lack of lateral or discal spines on the pronotum, the presence of two basal foveae on each elytron, and an elongate abdominal tergite 1(IV). *Arthromelodes* resembles *Batrisocenus* Raffray (also *Physomerinus* Jeannel and *Batriscenaulax* Jeannel) and *Batrisceniola* Jeannel in most external features (probably due to extensive homoplasy of characters), and under current definition may be separated from the former only by the aedeagus with a moderately developed basal capsule (capsule is strongly reduced in *Batrisocenus*; *e.g.*, Chandler 2001: 264), and from the latter by the lack of a median bunch of erect setae on tergite 4(VII) (bunched setae are present on tergite 4 in both sexes of *Batrisceniola*; *e.g.*, Yin and Li 2014). Within *Arthromelodes*, species are usually easy to determine thanks to the distinct sexual modifications of the male metatibiae (usually present), and of abdominal tergites 1(IV) and 4(VII).

A large series of pselaphine beetles collected in Taiwan by Dr. Wen-I Chou was recently sent to me for identification. A majority of the specimens belong to four species: *Cratna abdominalis* Löbl (347 specimens), *Taiwanophodes minor* Hlaváč (282 specimens), *Pselaphodes linae* Yin & Li (123 specimens), and *Batraxis splendida* Nomura (31 specimens); the rest include a *Labomimus* sp. (36 specimens), a *Batrisoschema* sp. (2 specimens), and a new species of *Arthromelodes* (8 specimens), representing the first record of this genus in China, which is described and compared to similar congeners here.

## Materials and methods

**Figure 1.**
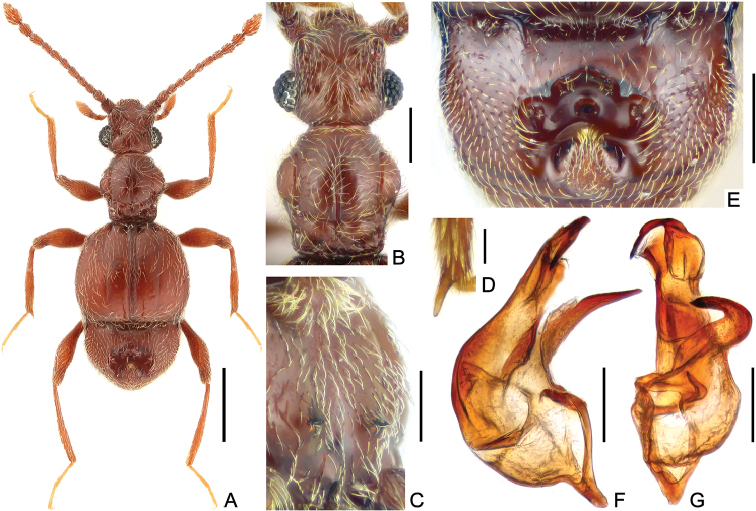
*Arthromelodes choui* sp. n., male. **A** habitus **B** head and pronotum **C** metaventrite, showing laminar projections at the middle **D** apex of mesotibia **E** tergite 1(IV), showing abdominal modification **F–G** Aedeagus, in lateral (**F**), and ventral (**G**) view. Scale bars: 0.5 mm (**A**); 0.2 mm (**B**, **E**); 0.1 mm (**C, F–G**); 0.05 mm (**D**).

The type series is deposited in the Insect Collection of the Shanghai Normal University, Shanghai, China (**SNUC**), and the National Museum of Natural Science, Taichung, Taiwan (**NMNS**). The collecting data of the material are quoted verbatim; information not included on the label is placed in parentheses. Following Chandler (2001), the abdominal tergites and sternites are given Arabic numerals for visible sclerites, and Roman numerals indicate the morphological position. Habitus image (Fig. 1A) was taken using a Canon 5D Mark III camera in conjunction with a Canon MP-E 65 mm f/2.8 1–5× Macro Lens, and a Canon MT-24EX Macro Twin Lite Flash was used as light source. Images of the morphological details (Fig. 1B–G) were produced using a Canon G9 camera mounted on an Olympus CX31 microscope under transmitted or reflected light. Zerene Stacker version 1.04 was used for image stacking. All images were edited and grouped in Adobe Photoshop CS5 Extended.

## Taxonomy

### Arthromelodes choui sp. n.

Taxon classificationAnimaliaColeopteraStaphylinidae

http://zoobank.org/619D8A25-690D-472A-9346-82C1E465F684

Fig. 1

#### Type material

(8 specimens)**. Holotype: CHINA**: ♂: ‘Taiwan, Taitung County (台东县), Haiduan Township (海端乡), Lidao (利稻), 23°10'55"N, 120°57'53"E, 1150 m, 24.iii.2017, light trap, Chou Wen-I leg.’ (in SNUC). **Paratype: CHINA**: 7 ♂♂, same collecting data as the holotype (5 in SNUC, 2 in NMNS).

#### Diagnosis of male.

Body length slightly more than 2 mm; metaventrite with pair of laminar projections; abdominal tergite 1(IV) with large cavity at posterior half, lateral setiferous patches composed of short setae and weakly demarcated; legs lacking modifications, except mesotibia with distinct apical spur; aedeagus with median lobe strongly curved rightwards apically, dorsal lobe erect, and strongly narrowed and curved downwards at apex.

#### Description.

Male (Fig. 1A). Length 2.09–2.22 mm; body reddish-brown, maxillary palpi and tarsi lighter. Head and pronotum (Fig. 1B) sparsely punctate. Head slightly wider than long, length from anterior margin of clypeus to head base 0.40–0.42 mm, width across compound eyes 0.44–0.47 mm; each eye composed of about 30 facets. Antennal clubs loosely formed by apical three antennomeres. Pronotum about as long as wide, length along midline 0.46–0.48 mm, maximum width 0.44–0.48 mm; lateral margins rounded at middle, constricted and nearly parallel at basal 1/3. Elytra wider than long, length along suture 0.66–0.72 mm, maximum width 0.77–0.83 mm; shallow discal striae reaching past 3/4 of elytral length; marginal sulcus complete; with slight denticle at humeral angle. Metaventrite with one pair of laminar projections (Fig. 1C) at middle. Mesotrochanter slightly protuberant at ventral margin; mesotibia with distinct apical spur (Fig. 1D). Abdomen slightly narrower than elytra, length of dorsally visible part posterior to elytra along midline 0.57–0.60 mm, maximum width 0.65–0.68 mm; tergite 1(IV) (Fig. 1E) much longer than 2–4 (V–VII) combined, deeply and broadly concaved at posteromedian portion, anterior margin of cavity angularly protruding posteriorly, with two pairs of secretory setae, elongate setae along lateral margins pointed posteromedially, bottom of cavity glabrous, with two pairs of secretory setae at middle and at sides, with one large nodule located posterior of cavity, setiferous lateral patches composed of short setae and weakly demarcated. Aedeagus (Fig. 1F–G) asymmetric, length 0.37 mm; median lobe strongly curved rightwards apically, capsule with distinct basoventral projection; parameres fused and reduced to ventral membrane; dorsal lobe erect, strongly narrowed and curved downwards at apex.

Female. Unknown.

#### Comments.

Using the key in Nomura (1991), *Arthromelodes choui* would key out at couplet 9 with *A. sinuatipes* Nomura and *A. aizuanus* Nomura. These three species share the presence of a cavity on male tergite 1(IV), a nodule placed posterior to the cavity, as well as a pair of setiferous patches lateral to the cavity. *Arthromelodes choui* may be readily separated from *A. sinuatipes* by the less laterally expanded margins of tergite 1, the less demarcated setiferous patches, the sinuate anterior margin of tergal cavity, and the much less transverse basal capsule of the aedeagus; and from *A. aizuanus* by the much larger abdominal cavity, the broader and less demarcated setiferous patches, and much shorter basoventral projection of the aedeagus. A similar tergal cavity and nodule are also present in *A. dilatatus* (Raffray) (with four subspecies), but this species can be easily characterized by the distinct lateral expansions at sides of tergite 1(IV), while the new species lacks such structures. Arai (2002) described *A. watanabei* Arai from Honshu, Japan, and this species may be separated from *A. choui* by the cavity on tergite 1 being much smaller and shallower, and by the form of the aedeagus (Arai, 2002: figs 1, 6–7). According to the description and figures in Jeannel (1954: 249), *A. choui* may be well-separated from *A. carieri* Jeannel (type species of *Arthromelodes*) from Vietnam again by the different structures of the male tergal cavity and aedeagus.

#### Distribution.

Southern China: Taiwan.

#### Etymology.

The specific epithet is dedicated to my friend Wen-I Chou, a Taiwanese specialist of Cerambycidae, who collected the type series of the new species.

## Supplementary Material

XML Treatment for Arthromelodes choui

## References

[B1] Arai S (2002) A new species of the genus *Arthromelodes* (Coleoptera, Staphylinidae, Pselaphinae) from Kanto District, central Japan. Special Bulletin of the Japanese Society of Coleopterology 5: 275–279.

[B2] Chandler DS (2001) Biology, morphology and systematics of the ant-like litter beetles of Australia (Coleoptera: Staphylinidae: Pselaphinae). Memoirs on Entomology, International 15: 1–560.

[B3] Jeannel R (1954) Les Psélaphides de Madagascar. Mémoires de l’Institut Scientifique de Madagascar (E: Entomologie) 4: 139–344.

[B4] Jeannel R (1957) Sur quelques Psélaphides du Tonkin recueillis par le Père A. de Cooman. Revue Française d’Entomologie 24(1): 5–32.

[B5] Jeannel R (1958) Révision des Psélaphides du Japon. Mémoires du Muséum National d’Histoire Naturelle (A: Zoologie) 18: 1–138.

[B6] Jeannel R (1959) Révision des Psélaphides de l’Afrique intertropicale. Annales du Musée Royal du Congo Belge, Tervuren (Série 8: Sciences Zoologiques) 75: 1–742.

[B7] Nomura S (1991) Systematic study on the genus *Batrisoplisus* and its allied genera from Japan (Coleoptera, Pselaphidae). Esakia 30: 1–462.

[B8] Yin ZW Li LZ (2014) *Batrisceniola fengtingae* sp. nov., the first record of the genus in China (Coleoptera: Staphylinidae: Pselaphinae). Acta Entomologica Musei Nationalis Pragae 54: 233–236.

